# P-1033. Determinants of In-hospital Mortality in Patients with Central Line-Associated Bloodstream Infections

**DOI:** 10.1093/ofid/ofaf695.1229

**Published:** 2026-01-11

**Authors:** Denise Araujo, Anita Shallal, Jennifer Mclenon, Amanda Hagedorn, Abigail Ruby, Eman Chami, Geehan Suleyman

**Affiliations:** Henry Ford Health, Detroit, MI; Henry Ford Hospital, Detroit, Michigan; Henry Ford Health, Detroit, MI; Henry Ford Hospital, Detroit, Michigan; Henry Ford Health, Detroit, MI; Henry Ford Hospital, Detroit, Michigan; Henry Ford Health, Detroit, MI

## Abstract

**Background:**

Central line-associated bloodstream infections (CLABSIs) are serious hospital-acquired infections associated with significant morbidity and mortality. Although risk factors associated with CLABSIs have been described, characterization and outcome are limited.

**Table 1.**
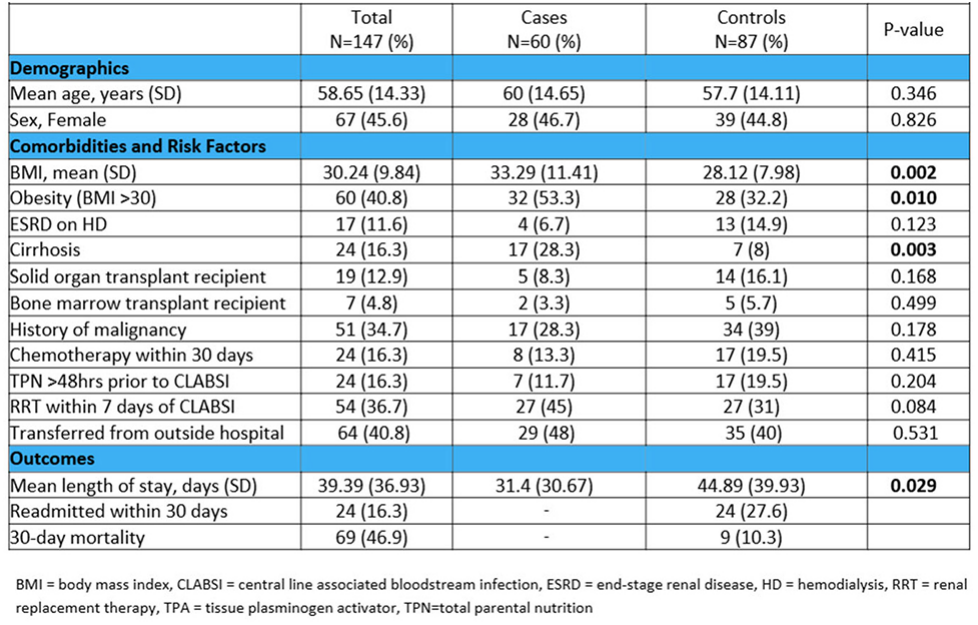
Clinical characteristics, risk factors, and outcomes of CLABSI patients with (cases) and without (controls) in-hospital mortality.

**Table 2.**
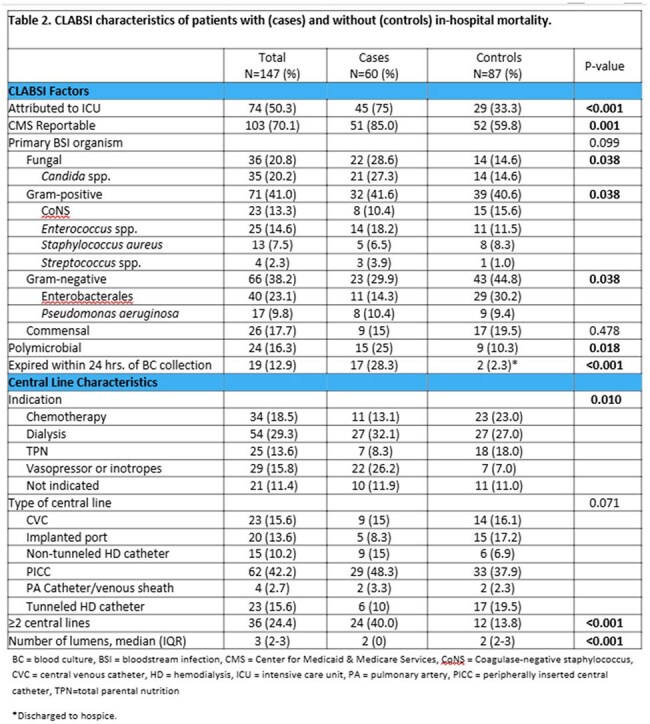
CLABSI characteristics of patients with (cases) and without (controls) in-hospital mortality.

**Methods:**

Retrospective cohort study of adult patients with (cases) and without (controls) in-hospital mortality (IHM) who were diagnosed with a CLABSI diagnosis between 1/2023 and 12/2024 at tertiary care center in Southeast Michigan. Patients meeting National Healthcare Safety Network (NHSN) CLABSI criteria were included. Comorbidities, CLABSI characteristics, risk factors and prevention practices, and outcomes were evaluated.

**Table 3.**
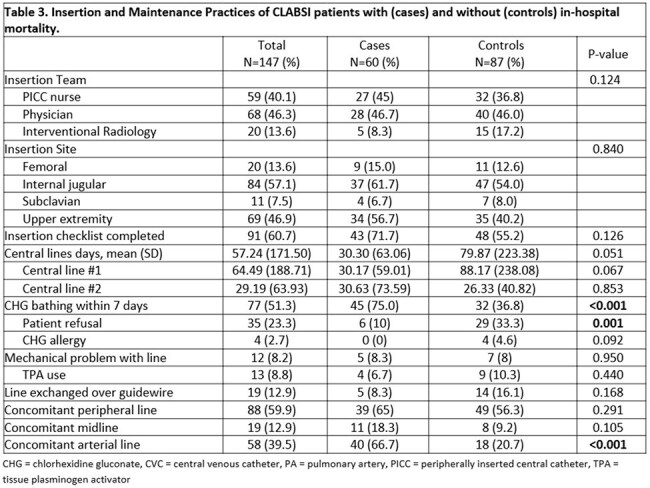
Insertion and Maintenance Practices of CLABSI patients with (cases) and without (controls) in-hospital mortality.

**Results:**

147 CLABSIs were evaluated, with 60 cases (41%) resulting in IHM (Table 1). Comorbidities were similar, except for obesity (53% vs. 32%, p=0.01) and cirrhosis (28% vs. 8%, p=0.03), which were more prevalent in cases. 11% of central lines (CL) were not indicated (Table 2). Use of vasopressor/inotrope use was higher in cases (26% vs 7.0%). CL days were exceedingly long in both groups. Gram-positive organisms were the most common cause of CLABSI, but Enterobacterales (23%) were the most frequently isolated pathogens. *Candida* spp. was more frequent in cases (27% vs 15%). Cases were significantly more likely to have polymicrobial infection (25% vs. 10%, p=0.018).

There were no differences in insertion or maintenance practices (Table 2), but cases were more likely to have concomitant arterial line, multiple lumens and CL, and higher CHG bathing compliance (Tables 2 and 3). CL line days were exceedingly long in both groups. ICU-attributable CLABSIs were significantly more prevalent in cases (75% vs. 33%, p< 0.001); almost a third died within 24 hrs. of blood culture (BC) collection.

**Conclusion:**

In our CLABSI cohort, patients with IHM were significantly more likely to have cirrhosis, polymicrobial BSI, multiple lines and lumens with prolonged duration, other indwelling devices and require ICU-level care. This study highlights the significant burden of IHM in patients with CLABSIs and underscores the importance of CL and BC stewardship in high-risk patients. Efforts should focus on targeted interventions to improve CLABSI prevention bundle compliance.

**Disclosures:**

All Authors: No reported disclosures

